# Effect of treatment modality and cerebral vasospasm agent on patient outcomes after aneurysmal subarachnoid hemorrhage in the elderly aged 75 years and older

**DOI:** 10.1371/journal.pone.0230953

**Published:** 2020-04-09

**Authors:** Keisuke Ido, Ryota Kurogi, Ai Kurogi, Kunihiro Nishimura, Koichi Arimura, Ataru Nishimura, Nice Ren, Akiko Kada, Ryu Matsuo, Daisuke Onozuka, Akihito Hagihara, So Takagishi, Keitaro Yamagami, Misa Takegami, Yasunobu Nohara, Naoki Nakashima, Masahiro Kamouchi, Isao Date, Takanari Kitazono, Koji Iihara, on behalf of the J-ASPECT Study Collaborators

**Affiliations:** 1 Department of Neurosurgery, Graduate School of Medical Sciences, Kyushu University, Fukuoka, Japan; 2 Department of Preventive Medicine and Epidemiology, National Cerebral and Cardiovascular Center, Suita, Japan; 3 Department of Clinical Research Management, National Hospital Organization Nagoya Medical Center, Nagoya, Japan; 4 Department of Health Communication, Graduate School of Medical Sciences, Kyushu University, Fukuoka, Japan; 5 Faculty of Advanced Science and Technology, Kumamoto University, Kumamoto, Japan; 6 Medical Information Center, Kyushu University Hospital, Fukuoka, Japan; 7 Department of Neurological Surgery, Okayama University Graduate School of Medicine, Okayama, Japan; 8 Department of Medicine and Clinical Science, Graduate School of Medical Science, Kyushu University, Fukuoka, Japan; Barrow Neurological Institute, UNITED STATES

## Abstract

**Objective:**

We sought to examine whether the effect of treatment modality and drugs for cerebral vasospasm on clinical outcomes differs between elderly and non-elderly subarachnoid hemorrhage (SAH) patients in Japan.

**Methods:**

We analyzed the J-ASPECT Study Diagnosis Procedure Combination database (n = 17,343) that underwent clipping or coiling between 2010 and 2014 in 579 hospitals. We stratified patients into two groups according to their age (elderly [≥75 years old], n = 3,885; non-elderly, n = 13,458). We analyzed the effect of treatment modality and anti-vasospasm agents (fasudil hydrochloride, ozagrel sodium, cilostazol, statin, eicosapentaenoic acid [EPA], and edaravone) on in-hospital poor outcomes (mRS 3–6 at discharge) and mortality using multivariable analysis.

**Results:**

The elderly patients were more likely to be female, have impaired levels of consciousness and comorbidity, and less likely to be treated with clipping and anti-vasospasm agents, except for ozagrel sodium and statin. In-hospital mortality and poor outcomes were higher in the elderly (15.8% *vs*. 8.5%, 71.7% *vs*. 36.5%).

Coiling was associated with higher mortality (odds ratio 1.43, 95% confidence interval 1.2–1.7) despite a lower proportion of poor outcomes (0.84, 0.75–0.94) in the non-elderly, in contrast to no effect on clinical outcomes in the elderly.

A comparable effect of anti-vasospasm agents on mortality was observed between non-elderly and elderly for fasudil hydrochloride (non-elderly: 0.20, 0.17–0.24), statin (0.63, 0.50–0.79), ozagrel sodium (0.72, 0.60–0.86), and cilostazol (0.63, 0.51–0.77). Poor outcomes were inversely associated with fasudil hydrochloride (0.59, 0.51–0.68), statin (0.84, 0.75–0.94), and EPA (0.83, 0.72–0.94) use in the non-elderly. No effect of these agents on poor outcomes was observed in the elderly.

**Conclusions:**

In contrast to the non-elderly, no effect of treatment modality on clinical outcomes were observed in the elderly. A comparable effect of anti-vasospasm agents was observed on mortality, but not on functional outcomes, between the non-elderly and elderly.

## Introduction

The incidence of subarachnoid hemorrhage (SAH) increases with advancing age [1,2].

The elderly patients with aneurysmal SAH (aSAH) have a greater risk of complications and poor outcomes than the risk in non-elderly patients due to worse clinical status on admission, less active management, and a higher frequency of comorbidity [2,3]. However, elderly patients with aSAH are increasingly receiving definitive treatment due to an increased proportion of those with premorbid high activities of daily living and recent progress of endovascular therapy [4]. Recently, endovascular treatment was established as a complementary treatment modality to neurosurgery, especially in the elderly and poor-grade patients with SAH [3].

Cerebral vasospasm and vasospasm-related cerebral infarction are serious complications, and important causes of death and dependency in patients with aSAH [5,6,7]. Previous studies have reported the effect of fasudil hydrochloride [8.^9^], ozagrel sodium [10], cilostazol [11], statin [12], eicosapentaenoic acid (EPA) [13] and edaravone [14], but not on the clinical outcomes, on cerebral vasospasm following aSAH mainly in the non-elderly population. The Consensus 2009 on the diagnosis and treatment of cerebral vasospasm in Japan reported that fasudil hydrochloride is the most commonly used prophylactic agent for symptomatic vasospasm. Further, edaravone is used most frequently for cerebral ischemia after aSAH in a real-world clinical practice in Japan [15]. However, the effects of these drug therapies for cerebral vasospasm and ischemia on the clinical outcome in elderly patients have not been elucidated.

Previous studies have shown that the clinical outcome of patients after aSAH is significantly different when the cutoff age was set at 75 years [2]. However, no previous papers have examined whether the comparative therapeutic effects exist in elderly patients aged ≥75 years of age. Therefore, we sought to examine whether the comparative effect of treatment modality and cerebral vasospasm agents exists on the clinical outcomes between non-elderly and elderly patients who were urgently hospitalized for aSAH between April 1, 2010, and March 31, 2014, using the nationwide DPC database (J-ASPECT Study) [16,17].

## Methods

### Ethics statement

This study was approved by the Kyushu University Institutional Review Board, which waived the requirement for individual informed consent.

### The DPC database

The DPC database is a mixed-case classification system linked with a lump-sum payment system that began in 2002 by the Ministry of Health, Labor, and Welfare of Japan [17]. In 2010, 1,388 acute care hospitals, representing about 60% of all hospital beds, adopted the DPC data system. Data on clinical practice can be obtained from the DPC database and the attending physician is responsible for clinical data entry for each patient. The DPC includes all patients admitted to participating hospitals. Compared with other registry databases, the strength of the DPC database is that it enables researchers to conduct nationwide studies of descriptive or analytical epidemiology in the real-world setting of clinical practice. The database includes data on the following elements: patient profile (i.e., age, sex, height, weight, smoking index); principal diagnoses (coded by the International Classification of Diseases and Injuries, 10th revision) and comorbidities at admission (coded similarly); complications after admission (coded similarly); and procedures, including surgery, medications and devices used during hospitalization, length of stay, discharge status [18]. Institutions using the DPC system encompass a wide variety of centers, including academic, large, urban, and rural hospitals [16].

### Sampling strategy

Participation in the J-ASPECT Study was voluntary, in collaboration with the Japan Neurosurgical Society and Japan Stroke Society. Of the 847 certified training institutions of the Japan Neurosurgical Society, 579 agreed to participate in the J-ASPECT Study. This cross-sectional survey utilized DPC discharge data from the participating institutions.

Computer software was developed to identify patients hospitalized for SAH from the de-identified discharge database using the International Classification of Diseases (ICD)-10 diagnosis codes related to SAH (I60.0–9). We extracted data from patients who had been urgently hospitalized for SAH from April 1, 2010 to March 31, 2014, from the Japanese DPC database. Patients with scheduled admission were excluded from this survey.

The DPC database includes the following data: patients’ age, sex, height, and weight; diagnoses; comorbidities on admission, including those based on the Charlson comorbidity index [19]; Brinkman index [20]; level of consciousness on admission according to the Japan Coma Scale (JCS); admission source; days between admission and treatment; in-hospital mortality; modified Rankin Scale (mRS) score at discharge; and procedures coded with Japanese original K-codes. The Charlson Comorbidity Index is a method of categorizing comorbidities of patients based on the International Classification of Diseases (ICD) diagnosis codes. A score of zero indicates that no comorbidities were found. The higher the score, the more likely the predicted outcome will result in mortality or higher resource use.

The extent of smoking was assessed based on the Brinkman index (daily number of cigarettes × years) [20]. The JCS is the most widely used grading scale for assessing impaired consciousness in Japan (**S1 Table**) [16,21]. The JCS represented the severity of conscious disturbance upon admission regardless of whether patients had subsequently undergone therapies for hydrocephalus (for example, ventriculostomy or CSF shunt).

### Statistical analysis

For statistical analysis, mRS score at discharge was dichotomized into 0–2 and 3–6. JCS score was treated as a categorical variable of 0, 1-, 2-, or 3-digit. Differences in patient demographics between non-elderly (<75 years old) and elderly (≥75 years old) groups were analyzed using the Wilcoxon rank sum test, t-test, and Chi-squared test. We used multivariable analysis to estimate the odds ratios (ORs) for in-hospital mortality and mRS score at discharge. In addition, subgroup analysis on the effect of cerebral vasospasm agents on clinical outcomes was conducted after excluding patients who presented in a deep coma to evaluate the effect of selection bias. In the subgroup analysis, the JCS score was categorized as 0, 1-, 2-digit, 100 and 200 combined, or 300. ORs and differences were adjusted using age, sex, height, weight, Brinkman index, JCS score, Charlson score, comorbidities (hypertension, diabetes mellitus, hyperlipidemia), and admission source. The analyses were performed using JMP^®^ Pro version 13 (SAS Institute Inc., Cary, NC, USA). P-values <0.05 were considered statistically significant.

## Results

### Patient demographics and outcomes

A total of 17,343 patients with SAH (<75 years old, n = 13,458; ≥75 years old, n = 3,885) at 579 institutions were identified based on ICD-10 codes. Table 1 shows the demographics of the both groups.

**Table 1:** Demographics and outcomes of the non-elderly group(<75y) and elderly group(≥75y).

	Non-elderly	Elderly	P-value
	n = 13,458	n = 3,885	
**Patients**			
Age, mean±SD	57.0±11.3	80.9±4.5	<0.01
Male, n (%)	4,957 (36.8)	498 (12.8)	<0.01
Height, mean cm±SD	159.9±8.8	150.8±7.5	<0.01
Weight, mean kg±SD	58.6±12.4	49.6±9.8	<0.01
Brinkman index, mean±SD	233.3±478.5	68.0±263.1	<0.01
**Comorbidities**			
Charlson score, mean n±SD	3.6±1.42	5.5±0.85	<0.01
Hypertension, n (%)	7,421 (55.1)	2231 (57.4)	0.012
Diabetes mellitus, n (%)	1,109 (8.2)	424 (10.9)	<0.01
Hyperlipidemia, n (%)	2,080 (15.5)	529 (13.6)	<0.01
**JCS score on admission**			<0.01
0, n (%)	3,233 (24.0)	624 (16.1)	
1-digit code, n (%)	3,902 (29.0)	1,156 (29.8)	
2-digit code, n (%)	2,968 (22.1)	914 (23.5)	
3-digit code, n (%)	3,355 (24.9)	1,191 (30.7)	
**Admission source**			
ambulance, n (%)	10,459 (77.7)	3,066 (78.9)	0.11
**Days between admission and treatment, mean±SD**	2.0±3.4	2.3±4.6	<0.01
**Treatment modality**			<0.01
Coiling, n (%)	3,621 (26.9)	1,369 (35.2)	
Clipping, n (%)	9,837 (73.1)	2,516 (64.8)	
**Drugs**			
Fasudil,hydrochloride n (%)	11,427 (84.9)	3,140 (80.8)	<0.01
Ozagrel sodium, n (%)	5,086 (37.8)	1,462 (37.6)	0.86
Cilostazol, n (%)	4,655 (34.6)	1,277 (32.9)	0.047
Statin, n (%)	3,995 (29.7)	1,160 (29.9)	0.84
Edaravone, n (%)	4,132 (30.7)	1,138 (29.3)	0.09
EPA, n (%)	2,233 (16.6)	558 (14.4)	<0.01
In-hospital mortality, n (%)	1,137 (8.5)	615 (15.8)	<0.01
Discharge mRS 3–6, n (%)	4,909 (36.5)	2,784 (71.7)	<0.01

Abbreviations: JCS, Japan Coma scale, mRS, modified Rankin Scale

Significant differences in the patient’s characteristics were observed between the non-elderly and elderly groups. In the elderly, the proportion of females was higher than in the non-elderly. The proportion of patients with impaired consciousness was significantly higher in the elderly group (e.g., comatose patients: non-elderly, 24.9%; elderly, 30.7%). In addition, the mean Charlson score was higher in the elderly group. Other comorbidities, such as hypertension and diabetes mellitus were higher in the elderly group, except for hyperlipidemia. The number of days between admission and treatment was slightly longer in the elderly group. The use of fasudil hydrochloride (84.9%, 80.8%, for non-elderly and elderly, respectively), cilostazol (34.6%, 32.9%), and EPA (16.6%, 14.4%) was more frequent in the non-elderly. In-hospital mortality (non-elderly, 8.5%; elderly, 15.8%) and the proportion of poor outcomes at discharge (mRS score of 3–6, 36.5% *vs*. 71.7%) were higher in the elderly group.

### Multivariable analysis

Table 2 shows the effect of the treatment modality and drugs for cerebral vasospasm on patient’s functional outcome (mRS 3–6 at discharge) in the non-elderly group and elderly group in multivariable analysis. In the non-elderly group, coiling and administration of fasudil hydrochloride, statin, and EPA was associated with a lower proportion of poor outcomes at discharge (mRS 3–6: coiling; 0.84, 0.75–0.94, fasudil hydrochloride; 0.59, 0.51–0.68, statin; 0.84, 0.75–0.94, EPA; 0.83, 0.72–0.94).

**Table 2:** Effect of the treatment modality and drugs on the poor outcome of patients (mRS3-6) with subarachnoid hemorrhage in multivariable analysis.

	Non-elderly			Elderly		
	OR	95% CI	P value	OR	95% CI	P value
**Treatment modality**						
Coiling	0.84	0.75–0.94	<0.01	0.88	0.73–1.06	0.19
**Drugs**						
Fasudil hydrochloride	0.59	0.51–0.68	<0.01	0.85	0.66–1.08	0.19
Ozagrel sodium	1.04	0.94–1.15	0.45	1.13	0.93–1.34	0.21
Cilostazol	0.91	0.82–1.01	0.07	1.00	0.83–1.21	1.00
Statin	0.84	0.75–0.94	<0.01	1.00	0.82–1.23	0.98
Edaravone	2.34	2.12–2.59	<0.01	2.33	1.89–2.86	<0.01
EPA	0.83	0.72–0.94	<0.01	0.80	0.63–1.01	0.07

ORs were adjusted by sex, age, JCS, height, weight, Brinkman index, Charlson score, comorbidities (hypertension, diabetes mellitus, hyperlipidemia), ambulance, days between admission and treatment, treatment modality and drugs (Cilostazol, Edaravone, EPA, Fasudil, Ozagrel, Statin).

Abbreviations: OR, Odds ratio; CI, confidence interval; EPA, eicosapentaenoic acid; mRS, modified Rankin Scale

In the elderly, no significant factors were associated with poor outcomes at discharge. In both elderly and non-elderly groups, those who were administered edaravone were associated with a higher proportion of poor outcomes (non-elderly; 2.34, 2.12–2.59, elderly; 2.33, 1.89–2.86).

Table 3 shows the effect of treatment modality and drugs for cerebral vasospasm on in-hospital mortality in the non-elderly and elderly groups in the multivariable analysis.

**Table 3:** Effect of the treatment modality and drugs on the in-hospital mortality of patients with subarachnoid hemorrhage in multivariable analysis.

	Non-elderly			Elderly		
	OR	95% CI	P value	OR	95% CI	P value
**Treatment modality**						
Coiling	1.43	1.20–1.70	<0.01	1.18	0.94–1.49	0.15
**Drugs**						
Fasudil hydrochloride	0.20	0.17–0.24	<0.01	0.33	0.25–0.42	<0.01
Ozagrel sodium	0.72	0.60–0.86	<0.01	0.79	0.62–1.01	0.06
Cilostazol	0.63	0.51–0.77	<0.01	0.61	0.47–0.79	<0.01
Statin	0.63	0.50–0.79	<0.01	0.63	0.48–0.85	<0.01
Edaravone	1.64	1.38–1.95	<0.01	1.25	0.98–1.59	0.07
EPA	0.80	0.60–1.05	0.11	0.91	0.64–1.31	0.62

ORs were adjusted by sex, age, JCS, height, weight, Brinkman index, Charlson score, comorbidities (hypertension, diabetes mellitus, hyperlipidemia), ambulance, days between admission and treatment, treatment modality and drugs (Cilostazol, Edaravone, EPA, Fasudil, Ozagrel, Statin).

Abbreviations: OR, Odds ratio; CI, confidence interval; EPA, eicosapentaenoic acid

In the non-elderly, administration of fasudil hydrochloride, ozagrel sodium, cilostazol and statin were associated with reduced in-hospital mortality (fasudil hydrochloride; 0.20, 0.17–0.24, ozagrel sodium; 0.72, 0.60–0.86, cilostazol; 0.63, 0.51–0.77, statin; 0.63, 0.50–0.79).

In the elderly, administration of fasudil hydrochloride, cilostazol and statin was associated with reduced in-hospital mortality (fasudil hydrochloride; 0.33, 0.25–0.42, cilostazol; 0.61, 0.47–0.79, statin; 0.63, 0.48–0.85), whereas the association between ozagrel sodium and in-hospital mortality was not present. Of note, the effect size of use of cilostazol and statin remained unchanged between non-elderly and elderly groups.

In the non-elderly, treatment with edaravone showed higher in-hospital mortality. In the elderly group, no factors were associated with increased in-hospital mortality.

### Subgroup analysis

The association between the level of consciousness on admission and the frequency of cerebral vasospasm agent use was evaluated by univariate (Table 4) and multivariable analysis (Table 5). Univariate analysis indicated that all drug treatments were less frequently administered in comatose patients; however, multivariable analysis revealed that only fasudil hydrochloride and cilostazol were administered less frequently in patients in deep coma (JCS 300), after adjusting for age, sex, and comorbidity (Table 5). After excluding patients in deep coma, similar effects were observed on the clinical outcomes after aSAH, except for the effect of EPA administration on poor outcomes after aSAH (Tables 6 and 7).

**Table 4:** Association between level of consciousness on admission and frequency of use of cerebral vasospasm agents in univariate analysis.

	JCS 0	JCS 1-digit	JCS 2-digit	JCS 100 and 200	JCS 300	P value
	n = 3,857	n = 5,058	n = 3,882	n = 3,126	n = 1,420	
Fasudil hydrochloride, n (%)	3239 (84.0)	4313 (85.3)	3403 (87.7)	2557 (81.8)	1055 (74.3)	<0.01
Ozagrel sodium, n (%)	1376 (35.7)	1965 (38.9)	1568 (40.4)	1177 (37.7)	462 (32.5)	<0.01
Cilostazol, n (%)	1319 (34.2)	1814 (35.9)	1396 (36.0)	983 (31.5)	420 (29.6)	<0.01
Statin, n (%)	1171 (30.4)	1547 (30.6)	1210 (31.2)	856 (27.4)	371 (26.1)	<0.01
Edaravone, n (%)	1123 (29.1)	1609 (31.8)	1205 (31.0)	940 (30.1)	393 (27.7)	<0.01
EPA, n (%)	632 (16.4)	858 (17.0)	652 (16.8)	457 (14.6)	192 (13.5)	<0.01

Abbreviations: EPA, eicosapentaenoic acid

**Table 5:** Association between level of consciousness on admission and frequency of use of cerebral vasospasm agents in multivariable analysis.

		OR	95% CI	P value
Fasudil hydrochloride	JCS 1-digit	1.13	1.01–1.27	0.04
	JCS 2-digit	1.39	1.22–1.58	< .01
	JCS 100 and 200	0.90	0.80–1.03	0.12
	JCS 300	0.58	0.50–0.68	< .01
Ozagrel sodium	JCS 1-digit	1.14	1.05–1.24	0.00
	JCS 2-digit	1.21	1.11–1.33	< .01
	JCS 100 and 200	1.10	0.99–1.21	0.06
	JCS 300	0.88	0.78–1.01	0.06
Cilostazol	JCS 1-digit	1.10	1.01–1.20	0.04
	JCS 2-digit	1.10	1.00–1.21	0.04
	JCS 100 and 200	0.93	0.84–1.03	0.15
	JCS 300	0.85	0.74–0.97	0.02
Statin	JCS 1-digit	1.05	0.95–1.16	0.31
	JCS 2-digit	1.10	0.99–1.22	0.07
	JCS 100 and 200	1.01	0.90–1.13	0.87
	JCS 300	0.97	0.84–1.12	0.70
Edaravone	JCS 1-digit	1.14	1.04–1.25	<0.01
	JCS 2-digit	1.10	1.00–1.21	0.05
	JCS 100 and 200	1.05	0.95–1.17	0.32
	JCS 300	0.94	0.82–1.08	0.38
EPA	JCS 1-digit	1.08	0.96–1.21	0.19
	JCS 2-digit	1.08	0.96–1.23	0.20
	JCS 100 and 200	0.99	0.87–1.14	0.92
	JCS 300	0.92	0.77–1.10	0.37

ORs were adjusted by sex, age, JCS, Charlson score, comorbidities (hypertension, diabetes mellitus, hyperlipidemia). JCS 0 was used as the reference.

Abbreviations: OR, Odds ratio; CI, confidence interval; EPA, eicosapentaenoic acid

**Table 6:** Effect of the treatment modality and drugs on the poor outcome (mRS3-6) of subarachnoid hemorrhage after excluding patients in deep coma in multivariable analysis.

	Non-elderly			Elderly		
	OR	95% CI	P value	OR	95% CI	P value
**Treatment modality**						
Coiling	0.82	0.73–0.92	<0.01	0.89	0.73–1.08	0.24
**Drugs**						
Fasudil hydrochloride	0.64	0.55–0.74	<0.01	0.86	0.67–1.11	0.26
Ozagrel sodium	1.04	0.94–1.16	0.39	1.11	0.92–1.34	0.28
Cilostazol	0.94	0.84–1.04	0.24	1.03	0.85–1.26	0.75
Statin	0.85	0.76–0.96	<0.01	1.02	0.83–1.25	0.88
Edaravone	2.42	2.18–2.69	<0.01	2.40	1.95–2.97	<0.01
EPA	0.81	0.71–0.93	<0.01	0.74	0.58–0.95	0.02

ORs were adjusted by sex, age, JCS, height, weight, Brinkman index, Charlson score, comorbidities (hypertension, diabetes mellitus, hyperlipidemia), ambulance, days between admission and treatment, treatment modality and drugs (cilostazol, edaravone, EPA, fasudil, ozagrel, statin).

Abbreviations: OR, Odds ratio; CI, confidence interval; EPA, eicosapentaenoic acid; mRS, modified Rankin Scale

**Table 7:** Effect of the treatment modality and drugs on the in-hospital mortality of subarachnoid hemorrhage after excluding patients in deep coma in multivariable analysis.

	Non-elderly			Elderly		
	OR	95% CI	P value	OR	95% CI	P value
**Treatment modality**						
Coiling	1.28	1.05–1.57	0.01	1.20	0.94–1.55	0.15
**Drugs**						
Fasudil hydrochloride	0.22	0.18–0.27	<0.01	0.35	0.26–0.46	<0.01
Ozagrel sodium	0.75	0.62–0.92	<0.01	0.81	0.62–1.05	0.11
Cilostazol	0.69	0.55–0.86	<0.01	0.60	0.45–0.80	<0.01
Statin	0.66	0.51–0.86	<0.01	0.62	0.46–0.85	<0.01
Edaravone	1.80	1.49–2.17	<0.01	1.26	0.97–1.63	0.09
EPA	0.82	0.60–1.10	0.19	1.03	0.70–1.49	0.89

ORs were adjusted by sex, age, JCS, height, weight, Brinkman index, Charlson score, comorbidities (hypertension, diabetes mellitus, hyperlipidemia), ambulance, days between admission and treatment, treatment modality and drugs (cilostazol, edaravone, EPA, fasudil, ozagrel, statin).

Abbreviations: OR, Odds ratio; CI, confidence interval; EPA, eicosapentaenoic acid

## Discussion

To the best of our knowledge, this is the first study to report that the therapeutic effects of clipping, coiling, and drug administration for cerebral vasospasm are different between non-elderly and elderly patients.

### Effects of treatment modality on clinical outcome after aSAH

In the non-elderly, an increased in-hospital mortality of coiling compared with clipping was observed. This effect was not present in our elderly group (age, ≥75 years). In contrast, better functional outcomes after coiling were noted in the non-elderly when compared with the elderly group. Previously, we have shown that in-hospital mortality rates in clipping patients after aSAH in Japan are significantly lower than those in the other countries, while in-hospital mortality rates after coiling are comparable [22]. The present study showed that the relative therapeutic advantage of clipping over coiling with respect to in-hospital mortality may decrease in patients ≥75 years of age.

Although coiling was used more frequently in the elderly when compared with the non-elderly, the proportion of patients who underwent coiling in both groups were similar to that of two previous studies [23,24] that were conducted relatively soon after the publication of the International Subarachnoid Aneurysm Trial (ISAT) [25]; however, our rates were lower than a recent US report [26].

Better functional outcomes for coiling in non-elderly patients in this study are consistent with those in previous reports. For example, the long-term results of the ISAT showed high independent survival rates in the coiling group (OR 1.34, 95% CI 1.07–1.67) [27]. In addition, recent nationwide database studies in the U.S. have revealed better functional outcomes for patients with aSAH who underwent coiling (clipping, 42%; coiling, 36%; OR 1.32 95% CI 1.12–1.56), although no significant differences were observed in mortality between the groups (clipping, 12%; coiling, 13%; OR 0.94 95% CI 0.73–1.21) [26]. The mean age of patients with aSAH in these previous reports is similar to that of the non-elderly group in the present study. This suggests that the widely recognized benefit of coiling in terms of better outcomes for aSAH is not generalizable to the patients aged ≥75 years. A recent study reported the effect of perioperative aneurysm rebleeding on aSAH patient outcomes [28]. Additional studies are required to determine the effect of unmeasured confounding factors.

The lack of an association between the treatment modality and outcomes in the elderly aSAH patients is consistent with the findings of previous studies focusing on the effects of coiling of ruptured aneurysms in elderly patients that used a different cutoff of definition for elderly. The ISAT cohort included 278 aSAH patients aged ≥65 years and reported that treatment modality did not affect functional outcome (independent functional outcomes coiling 60.1% versus clipping 56.1%) [3]. Proust et al. have analyzed aSAH patients aged ≥70 years who were treated from 1997–2007. Although age ≥75 years, poor initial grade, and occurrence of ischemia are associated with poor prognosis, the treatment modality did not affect patient’s outcomes [29].

### Effects of drug therapy for cerebral vasospasm on clinical outcome after aSAH

To the best of our knowledge, this is the first report that demonstrated age-related differences in the effects of drug therapies for cerebral vasospasm and ischemia on clinical outcomes after aSAH. In the US and Europe, nimodipine is effective for the prevention of delayed cerebral ischemia (DCI) and reduces the risk of poor clinical outcomes [30,31]. Further, it is recommended in the American Heart Association/American Stroke Association guidelines [32]. In Japan, nimodipine is not approved, and fasudil hydrochloride [8,9], ozagrel sodium [10], statins [12], cilostazol [11], edaravone [14], and EPA [13] are used to suppress the occurrence of symptomatic cerebral vasospasm and decreases exacerbation of neurological symptoms due to cerebral vasospasm following aSAH. A meta-analysis measuring the effect of pharmaceutical treatment on vasospasm, delayed cerebral ischemia, and clinical outcome in aSAH patients has demonstrated that pharmaceutical treatments for cerebral vasospasm significantly decrease the incidence of vasospasm, but not of poor outcomes [33]. In addition, the effects of other cerebral vasospasm agents on clinical outcomes after aSAH were reported and these results supported the lack of association between vasospasm and clinical outcomes. For example, a prospective randomized study has reported that the effect of cilostazol is associated with decreased risk of symptomatic vasospasm, cerebral infarction, but not poor outcomes [34]. In addition, simvastatin has no effect on favorable outcomes and mortality [35]. A multicenter randomized study showed that EPA reduces symptomatic vasospasm and cerebral infarction; however, it does not improve functional outcomes [13].

For cerebral ischemia, edaravone, which has limited clinical significance for acute ischemic stroke [36], was associated with a trend toward reduced poor outcomes caused by cerebral vasospasm after aSAH [14]. Notably, the mean ages of participants in these previous studies ranged from 53–62 years [14,34,8–13], which is similar to the mean age of the non-elderly group in the present study (57 years).

Thus, an important finding of this study is that in non-elderly patients, the use of the majority of these agents is associated with short-term poor outcomes and in-hospital mortality after aSAH. However, this study did not evaluate the effect of drug treatment on vasospasm. In contrast, in the elderly group, no drugs, except for EPA, were associated with a decreased incidence of poor outcome (mRS 3–6) at discharge. Further, administration of fasudil hydrochloride, cilostazol, and statin decreased in-hospital mortality.

### Differences in the therapeutic effect of the drugs based on the age

Some characteristics of elderly patients with aSAH may account for the difference in the therapeutic effect of the drugs that we have shown. Elderly patients with aSAH are more likely to be associated with a severe neurological status at onset due to the vulnerability of the brain, underlying comorbidity, and higher risk of systemic complications, such as pneumonia. Therefore, it is possible that the therapeutic effect of anti-vasospasm drugs may be masked because of worse functional state at discharge due to causes other than DCI [29]. However, the present study clearly showed the therapeutic effects of anti-vasospasm drug treatment on decreased in-hospital mortality. This may be due to a decrease in severe cerebral vasospasm and accompanying DCI. This suggests that aggressive drug treatment for cerebral vasospasm is important after aSAH in elderly patients. Patient cause of death is unknown in this study; therefore, further studies are required to elucidate the disparity between the results on functional outcome and in-hospital mortality.

The use of edaravone was significantly associated with poor outcomes in both groups and increased in-hospital mortality in the non-elderly group. In Japan, edaravone is not used as a prophylactic agent for cerebral vasospasm; however, it is administered for cerebral infarction in clinical practice [15]. Therefore, use of edaravone in this study may be considered as a surrogate marker of the development of cerebral infarction associated with poor patient outcomes. Therefore, it is difficult to determine the independent effect of edaravone on the cerebral vasospasm.

### Selection bias for using cerebral vasospasm agents for aSAH

The association between the use of cerebral vasospasm agents and clinical outcomes shown in this study deserves some mention. One may argue that the association between the use of cerebral vasospasm agents and clinical outcomes, especially in-hospital mortality, may be explained by selection bias introduced by the use of such drugs for patients with better level of consciousness. The use of cerebral vasospasm agents may be withheld for poor grade aSAH patients, especially for patients in deep coma. In line with this, fasudil hydrochloride and cilostazol, but not other agents, were administered less frequently in patients in deep coma in this study. In contrast, there were administered to a significant proportion of patients in deep coma.

In the post-ISAT era, early and aggressive treatment results in a significant improvement in the survival rate of comatose patients with Hunt and Hess Grade V SAH when compared with earlier periods [37]. This indicates that younger age and bilateral intact corneal reflexes are independent predictors of favorable outcomes. In contrast, a recent study from Canada and Europe reported that the decision to withdraw life support is the major reason of death of patients with aneurysmal SAH for a large majority of the patients with aSAH [38]. In Japan, the proportion of end-of-life decisions in Japan is reportedly much lower than in other countries [39]. In addition, only patients who underwent definitive treatment for aSAH with a comparable interval between admission and clipping/coiling were included in this study. The overall effect size of drug administration may be overestimated, particularly for fasudil hydrochloride and cilostazol, due to their reduced administration in the patients in deep coma; however, a similar association between the use of such agents and clinical outcomes remained after excluding patients in deep coma in this study. Taken together, these results suggest that there is a reduced likelihood of a significant selection bias effect. Nonetheless, prospective studies should be conducted in the future to determine the causal relationship between the drug administration and clinical outcomes.

### Future perspective

Recently, patient-reported outcomes (PROMs) have attracted increased attention worldwide. PROMs provide additional valuable information when compared with the mRS score for patients with stroke in an ambulatory setting [40]. The effects of treatment modality and vasospasm agents on clinical outcomes after aSAH indicated the importance of choosing the correct treatment plan for elderly patients with aSAH. This should take into account their long-term quality of life [41] and/or SAH specific outcome [42] to promote value-based medicine for patients with aSAH.

## Limitations

Our study has several limitations. First, the DPC database lacks some important data, such as cause of death, the presence of symptomatic cerebral vasospasm, and the presence of DCI. This makes difficult to determine whether the treatment effect is due to prevention and suppression of the spasm. The DPC database also lacks detailed aneurysm characteristics. Confounders specific to Japan may underlie the differences in outcomes compared with other countries. Second, as a measure of SAH severity, we used the JCS score rather than the Hunt and Hess grade or the World Federation of Neurological Surgeons (WFNS) scale. The JCS is widely used for the assessment of impaired consciousness in patients with aSAH in Japan. This test only assesses diminished consciousness; however, JCS score on admission shows the greatest effect patient outcomes [43]. In addition, JCS scores are correlated with the clinical outcomes [21]. Therefore, we believe that the JCS is an appropriate indicator of SAH severity. In addition, data on Fisher grade was not available. Third, end-of-life decisions are important factor affecting mortality rates; however, this information was not available in this study. Insights into the causes of death should be prospectively assessed to improve care and clinical outcomes, especially for elderly patients with aSAH [38]. Fourth, detailed information on the frequent and early use of endovascular treatment for cerebral vasospasm was not analyzed in this study [44]. A further study is required to determine the effect of unmeasured confounders.

## Conclusions

In contrast to the non-elderly group, there was no effect of treatment modality on aSAH patient outcomes in the elderly group (age ≥75 years). We found a comparable effect of treatment with cerebral vasospasm agents on in-hospital mortality between groups, Interestingly, there was a difference in functional outcomes at discharge between the non-elderly and elderly group. Further studies are required to determine any effect of unmeasured confounders.

## Supporting information

S1 Table Japan Coma Scale for grading impaired consciousness. (DOCX)

S2 Table Contributors all Contributors have been involved in collection of data. (DOCX)
