# Supplementary material for: Effect of treatment modality and cerebral vasospasm agent on patient outcomes after aneurysmal subarachnoid hemorrhage in the elderly aged 75 years and older
Source: PLoS One. 2020 Apr 9;15(4):e0230953. doi: 10.1371/journal.pone.0230953 (PMC7145106; doi:10.1371/journal.pone.0230953)
Supplement: S1 Table — (DOCX) [file pone.0230953.s001.docx]

**Supplementary Table S1.** Japan Coma Scale for grading impaired consciousness*

| Grade | Consciousness Level |
| --- | --- |
| 1-digit code | The patient is awake without any stimuli, and is: |
| 1 | Almost fully conscious |
| 2 | Unable to recognize time, place, and person |
| 3 | Unable to recall name or date of birth |
| 2-digit code | The patient can be aroused (then reverts to previous state after cessation of stimulation): |
| 10 | By easily by being spoken to (or is responsive with purposeful movements, phrases, or words)† |
| 20 | With a loud voice or shaking of shoulders (or is almost always responsive to very simple words like yes or no or to movements) |
| 30 | Only by repeated mechanical stimuli |
| 3-digit code | The patient cannot be aroused with any forceful mechanical stimuli, and: |
| 100 | Responds with movements to avoid the stimulus |
| 200 | Responds with slight movements, including decerebrate and decorticate posture |
| 300 | Does not respond at all except for changes in respiratory rhythm |

*"R" and "I" are added to the grade to indicate restlessness and incontinence of urine and feces, respectively: for example; 100-R and 30-RI.

†Criteria in parentheses are used in patients who cannot open their eyes for any reason
