# Supplementary material for: Effect of treatment modality and cerebral vasospasm agent on patient outcomes after aneurysmal subarachnoid hemorrhage in the elderly aged 75 years and older
Source: PLoS One. 2020 Apr 9;15(4):e0230953. doi: 10.1371/journal.pone.0230953 (PMC7145106; doi:10.1371/journal.pone.0230953)
Supplement: S2 Table — (DOCX) [file pone.0230953.s002.docx]

**Supplementary Table S2.** Contributors

All Contributors have been involved in collection of data.

| Hospitals | Responsible persons |
| --- | --- |
| Sapporo City General Hospital | Masayoshi Takigami |
| Nakamura Memorial Hospital | Kenji Kamiyama |
| Hokkaido University Neurosurgery | Kiyohiro Houkin |
| Sapporo Higashi Tokushukai Hospital | Shougo Nishi |
| Kashiwaba Neurosurgical Hospital | Tetsuyuki Yoshimoto, Sadao Kaneko |
| Nakamura Memorial South Hospital | Koji Oka |
| Takikawa Neurosurgical Hospital | Yusuke Nakagaki |
| Muroran City General Hospital | Hiroshi Ooyama |
| Asahikawa Medical University Hosipital | Kyousuke Kamada |
| Japanese Red Cross Asahikawa Hospital | Kenichi Makino |
| Nayoro City General Hospital | Naoki Tokumitsu, Kazuhiro Sako, Naoki Tokumitsu |
| Rumoi Central Clinic | Susumu Suzuki |
| Japanese Red Cross Kitami Hospital | Nozomi Suzuki |
| Dohtoh Neurosurgical Hospital | Teruo Kimura |
| Medical Corporation Meiseikai Abashiri Neurosurgical Rehabilitation Hospital | Naoto Izumi |
| Hokuto Hospital | Kazumi Nitta |
| Obihiro Kosei General Hospital | Masahumi Ootaki |
| Kushiro Rosai Hospital | Masanori Isobe |
| Hakodate Neurosurgical Hospital | Mikio Nishiya, Takaaki Yamazaki |
| Hakodate Shintoshi Hospital |  |
| Otaru Municipal Medical Center for Brain Cardiovascular and Mental Disorders | Syouji Mabuchi |
| Iwate Medical University | Kuniaki Ogasawara |
| Morioka Red Cross Hospital | Naohiko Kubo |
| Iwate Prefectural Isawa Hospital | Yukihiko Shimizu |
| Iwate Prefectural Iwai Hospital | Keiichi Saito |
| Iwate Prefectural Ofunato Hospital | Tatumi Yamanome |
| Iwate Prefectural Ninohe Hospital | Akinori Yabuta, Akira Suzuki |
| Tokyo Metropolitan Health and Medical Treatment Corporation Toshima Hospital |  |
| Nihon University Itabashi Hospital | Atsuo Yoshino |
| Juntendo University Nerima Hospital |  |
| Corporate Medical Association Shoikai Kasai Shoikai Hospital | Junichi Harashina |
| Inagi Municipial Hospital | Mitsuyuki Fujitsuka |
| National Hospital Organization Disaster Medical Center | Hiroyuki Masaoka |
| Tokyo West Tokushukai Hospital | Masaaki Takami |
| Higashiyamato Hospital | Hirotoshi Ohtaka |
| Kyorin University Hospital | Teruyuki Hirano, Yosiaki Shiokawa |
| Tokyo Metropolitan Health and Medical Treatment Corporation Tama-Hokubu Medical Center | Takaharu Okada |
| Japanese Red Cross Medical Center | Ichiro Suzuki |
| Tokyo Medical University Hospital | Michihiro Kohno, Jou Haraoka |
| Kawakita General Hospital |  |
| Makita General Hospital | Yoshinori Arai, Noriyoshi Kawamura |
| Omori Red Cross Hospital | Akira Isoshima, Masaharu Yasue |
| Nitobe Memorial Nakano General Hospital | Mitsuhiko Hokari, Takayoshi Kobayashi |
| NTT Medical Center Tokyo | Kensuke Kawai |
| Tokyo Medical And Dental University | Taketoshi Maehara |
| Juntendo University Hospital | Hajime Arai |
| Tokyo Women's Medical University | Takakazu Kawamata, Yoshikazu Okada |
| Tokyo Teishin Hospital | Makoto Noguchi |
| Saiseikai Central Hospital | Haruhiko Hoshino |
| Japan Community Health Care Organization Tokyo Takanawa Hospital | Hirofumi Hiyama |
| Tokyo Metropolitan Hiroo Hospital | Kensaku Yoshida |
| Kugayama Hospital | Mitsuyuki Fujitsuka |
| Kosei Chuo General Hospital | Osamu Utsugi |
| Tokyo Yamate Medical Center | Yasuaki Takeda |
| Matsudo City Hospital | Kouichi Tamaki, Hirohide Karasudani |
| Juntendo University Urayasu Hospital | Takao Urabe |
| Nippon Medical School Chiba Hokusoh Hospital | Shiro Kobayashi |
| Narita Red Cross Hospital | Michio Nakamura |
| Chiba Emergency Medical Center | Yorio Koguchi |
| Chiba Cerebral And Cardiovascular Center | Junichi Ono |
| Kimitsu Chuo Hospital | Sumio Suda |
| Kameda Medical Center | Hiromu Hadeishi, Toshio Fukutake |
| New Tokyo Hospital |  |
| Ciba Neurosurgical Clinic | Kenji Wakui |
| National Hospital Organization Chiba Medical Center | Hirokazu Tanno, Naoki Ishige |
| Tokyo Women's Medical University Yachiyo Medical Center | Takashi Ohasi |
| Keishunkai Medical Corporation Kobari General Hospital | Naoaki Sato |
| National Hospital Organization Toyohashi Medical Center | Hideki Sakai, Yasuaki Nishimura |
| Toyokawa City Hospital | Takayuki Watanabe, Takashi Matsumoto |
| Tosei General Hospital | Naoki Koketsu |
| Fujita Health University Hospital | Yuichi Hirose |
| Aichi Medical University Hospital | Manabu Doyu |
| Komaki City Hospital | Toshinori Hasegawa |
| Kasugai Municipal Hospital | Naoto Kuwayama, Shinichi Terao |
| Konan Kosei Hospital | Nobuhiko Mizutani |
| Nagoya Medical Center | Noriyuki Suzaki, Satoshi Okuda |
| Japanese Red Cross Nagoya Daini Hospital | Keizo Yasui, Yukio Seki, Yasuhiro Hasegawa |
| Japan Community Healthcare Organization Chukyo Hospital | Akira Ikeda |
| Meitetsu Hospital | Youtarou Takeuchi, Sigeki Ohara |
| Nagoya University Hospital | Yoshio Araki, Toshihiko Wakabayashi |
| Minato Medical Coop - Kyoritsu General Hospital | Hisashi Tanaka |
| Nagoya Memorial Hospital | Junpei Yoshimoto |
| Okazaki City Hospital |  |
| Anjo Kosei Hospital | Makoto Sugiura |
| Toyota Kosei Hospital | Ogura Koichiro |
| Kainan Hospital | Nozomu Kobayashi |
| Nishio Municipal Hospital | Toshio Yokoe |
| Hekinan Municipal Hospital |  |
| Sakura General Hospital |  |
| Tsushima City Hospital |  |
| Shiroyama Hospital | Kenichi Murao |
| National Hospital Organization Osaka Minami Medical Center | Tomonori Yamada |
| Kinki University Hospital | Amami Kato, Toshiho Ohtsuki |
| Osaka Neurological Institute | Akatsuki Wakayama |
| National Cerebral And Cardiovascular Center | Jun Takahashi, Hiroharu Kataoka |
| Osaka University Hospital | Toshiki Yoshimine |
| Sakai City Medical Center | Yoshikazu Nakajima |
| Baba Memorial Hospital | Hidehuku Gi |
| Bellland General Hospital | Ryunosuke Uranishi |
| Sakai Hospital Kinki University | Yusaku Nakamura |
| Fuchu Hospital | Kazunori Yamanaka, Kazumi Ohmori |
| Kishiwada Tokushukai Hospital | Hiroyuki Matsumoto, Yoshitugu Oiwa |
| Osaka Saiseikai Nakatsu Hospital | Yosihiko Uemura |
| Meisei Hospital | Hiroaki Fujiwara |
| Osaka City General Hospital | Yoshiyasu Iwai |
| Yodogawa Chrlstian Hospital | Masashi Morikawa |
| Osaka Kouseinenkin Hospital |  |
| Tane General Hospital | Kazuyuki Tane |
| Osaka National Hospital | Kazuo Hashikawa, Toshiyuki Fujinaka |
| Nipponbashi Neurosurgical Hospital | Shunichi Yoneda |
| Osaka Red Cross Hospital | Kohsuke Yamashita |
| Osaka Police Hospital |  |
| Tominaga Hospital | Masahiko Kitano, Shinsuke Tominaga |
| Suisyoukai Murata Hospital | Kazuhito Nakamura |
| Saiseikai Noe Hospital | Katsuhiko Kono |
| Osaka City University Graduate School of Medicine | Kenji Ohata |
| Kouzenkai Yagi Neurosurgical Hospital | Hirokatsu Taniguchi |
| Osaka General Medical Center | Takanori Hazama |
| Osaka Medical College | Toshihiko Kuroiwa, Yoji Tamura |
| Takatsuki General Hospital | Kazusige Maeno |
| Midorigaoka Hospital | Motohiro Arai |
| Hoshigaoka Koseinenkin Hospital |  |
| Kansai Medical University Takii Hospital | Masaaki Iwase |
| Matsushita Memorial Hospital | Kenji Hashimoto, Keisuke Yamada |
| Iryouhoujintokushukai Yaotokushukai Sougoubyouin | Takashi Turuno, Tsutomu Ichinose |
| Ishinkai Yao General Hospital | Shinichiro Kurokawa |
| Wakakusa Daiichi Hospital | Takeshi Matsuyama |
| Kawachi General Hospital |  |
| Hanwa Memorial Hospital | Toshiaki Fujita, Takamichi Yuguchi |
| Yuaikai Hospital | Yoshihumi Teramoto, Hiroto Kakita |
| Nagasaki University Hospital | Takayuki Matsuo, Tsuyoshi Izumo |
| Nagasaki Municipal Hospital |  |
| Juzenkai Hospital | Nobutoshi Ryu |
| Saiseikai Nagasaki Hospital | Wataru Haraguchi, Naoki Kitagawa |
| Department Of Neurosurgery Sasebo City General Hospital | Makio Kaminogo |
| Sasebo Chuo Hospital | Seisaburo Sakamoto |
| Sankoukai Miyazaki Hospital |  |
| Nagasaki Prefecture Shimabara Hospital | Yosiharu Tokunaga |
| Nagasaki Kawatana Medical Center | Ei-Ichirou Urasaki |
| Kumamoto University Hospital | Junichi Kuratsu |
| Kumamoto City Hospital | Akira Takada |
| Japanese Red Cross Kumamoto Hospital | Tadashi Terasaki |
| Saiseikai Kumamoto Hospital | Toru Nishi |
| Arao Municipal Hospital | Isao Fuwa, Hisami Ooshima |
| Japan Labour Health And Welfare Organization Kumamoto Rosai Hospital | Shigeo Yamashiro |
| Minamata City General Hospital And Medical Center | Makoto Yoshikawa, Hiromasa Tsuiki |
| JCHO Kumamoto General Hospital | Kazunari Koga |
| Uki General Hospital | Hiroshi Egami |
| Nagatomi Neurosurgical Hospital | Hirofumi Nagatomi |
| Tenshindo Hetsugi Hospital | Tadao Kawamura |
| Almeida Memorial Hospital | Makoto Goda |
| Oita Prefectural Hospital | Yu Takeda |
| NHO Beppu Medical Center | Kunihiko Mitsuo |
| JCHO Nankai Medical Center | Takamitu Hikawa, Masaki Morisige, Yuu Takeda |
| Oita Oka Hospital | Yutaka Yamaguchi |
| Seiwakai Wada Hospital | Shiro Miyata, Shunro Uchinokura |
| Junwakai Memorial Hospital | Tomokazu Goya |
| University Of Miyazaki Hospital | Hideo Takeshima |
| Fujimoto General Hospital | Kazutaka Yatsushiro |
| Miyakonojo Medical Association Hospital | Hajime Ohta |
| Imakiire General Hospital | Tatsui Nagadou |
| Kagoshima City Hospital | Kazuho Hirahara |
| Kouseikai Obara Hospital | Souichi Obara |
| Izumi　Generai Medical Center | Hiroshi Seto |
| Kagoshima Prefectural Kanoya Medical Center | Shunichi Tanaka |
| Tokuda Neurosurgical Hospital | Koichi Moroki |
| Kagoshima University Graduate School Of Medicine And Dental Sciences | Kazunori Arita |
| University Of The Ryukyus Hospital | Shogo Ishiuchi |
| Okinawa Prefectural Hokubu Hospital | Toshimitsu Uchihara |
| Urasoe General Hospital | Susumu Mekaru |
| Okinawa Prefectural Nanbu Medical Center And Children's Medical Center | Tomoaki Nagamine |
| Naha City Hospital | Naoki Tomiyama, Jin Momoji |
| Okinawa Miyako Hospital | Satoshi Yamamoto |
| Okinawa Kyodo Hospital | Koji Idomari, Atushi Kimoto |
| Nanbu Tokushukai Hospital | Tsutomu Kadekaru |
| Okinawa Prefectural Yaeyama Hospital | Hirosi Syamoto |
| Niigata City General Hospital | Osamu Sasaki |
| Shinrakuen Hospital | Makoto Minagawa |
| Kuwana Hospital |  |
| Niigata University Medical & Dental Hospital | Yukihiko Fujii |
| Niigata Cancer Center Hospital | Hideaki Takahashi |
| Niigata Minami Hospital |  |
| Niigata Neurosurgical Hospital | Kiyoshi Onda, Hiroyuki Arai |
| Nagaoka Chuo General Hospital | Shigekazu Takeuchi |
| Tachikawa General Hospital | Hiroshi Abe |
| Saito Memorial Hospital | Osamu Fukuda |
| Niigata Tokamachi Hospital | Mitsuo Kouno |
| Niigata Prefectural Central Hospital | Tetsuro Tamura |
| Itoigawa General Hospital |  |
| Saiseikai Toyama Hospital | Yukio Horie, Michiya Kubo |
| Toyama Prefectural Central Hospital | Hiroaki Hondo |
| Toyama City Hospital | Tadao Miyamori |
| Himi Municipal Hospital | Hisashi Takada |
| Tonami General Hospital | Toru Masuoka |
| Shinseikai Toyama Hospital |  |
| Kaga City Hospital | Naoki Shirasaki |
| Komatsu Municipal Hospital | Hisashi Nitta |
| Yawata Medical Center | Makoto Kimura, Yasuo Katsuki |
| Ishikawa Prefectural Central Hospital | Yutaka Hayashi, Hisato Minamide, Shigeru Munemoto |
| Kanazawa Medical Univercity | Shunsuke Shiraga |
| National Hospital Organization Kanazawa Medical Center | Kiyonobu Ikeda |
| Kanazawa University | Mitsutoshi Nakada, Yutaka Hayashi |
| Kanazawa Neurosurgical Hospital | Syuji Sato |
| Japanese Red Cross Fukui Hospital | Taketo Hatano |
| University Of Fukui Hospital | Osamu Yamamura |
| Fukui Social Insurance Hospital | Masanori Kabuto |
| Hayashi Hospital | Takahiro Sakuma, Jyunya Hayashi |
| Tannan Regional Medical Center |  |
| University Of Yamanashi | Hiroyuki Kinouchi |
| Yamanashi Prefectural Central Hospital | Hidehito Koizumi |
| Yamanasi Kosei Hospital | Mikito Uchida |
| Fujiyoshida Hospital | Syougo Imae |
| Yamanashi Redcross Hospital | Hiroshi Ozawa |
| Juzen General Hospital |  |
| Saiseikai Imabari Hospital | Osamu Nishizaki |
| Ehime Prefectural Imabari Hospital | Manabu Fujita |
| Matsuyama Shimin Hospital | Masakazu Suga |
| Ehime Prefectural Central Hospital | Shinji Iwata, Kanehisa Kohno |
| Ehime University School of Medicine | Takeharu Kunieda |
| Uwajima City Hospital | Kiichiro Zenke |
| Katagi Neurological Surgery | Mutsuo Fujisawa |
| Okinawatokushuukai Uwajima Tokushukai Hospital | Hiromichi Sadashima |
| Izumino Hospital | Hikaru Mizobuchi |
| Chikamori Hospital (Chikamori Health Care Group) | Satoru Hayashi |
| Kochi Health Sciences Center | Masanori Morimoto |
| Japanese Red Cross Kochi Hospital | Takeshi Kohno |
| Kochi Medical School | Tetsuya Ueba |
| Kochi Prefectural Hata Kenmin Hospital | Hiroyuki Nishimura |
| Medical Corporation Usuikai Tano Hospital | Naoki Ikawa |
| Kagawa Prefectural Central Hospital | Yuzo Matsumoto |
| Takamatsu Municipal Hospital | Seiji Kannuki |
| Takamatsu Red Cross Hospital | Masahiro Kagawa |
| Osaka Neurosurgical Hospital | Naoki Hayashi |
| Kagawa University Faculty Of Medicine | Takashi Tamiya, Atsushi Shindo |
| Kagawa Rosai Hospital | Kimihiro Yoshino |
| Kaisei Hospital |  |
| Mitoyo General Hospital | Tetsuya Masaoka |
| Kokura Memorial Hospital | Ichiro Nakahara |
| Steel Memorial Yawata Hospital | Akira Nakamizo |
| Fukuokaken Saiseikai Yahata General Hospital | Yuji Okamoto |
| Fukuoka Shin Mizumaki Hospital | Shigenari Kin |
| Obase Hospital | Haruki Takahashi |
| Fukuoka Kieikai Hospital | Satoshi Suzuki |
| Kyushu University Hospital | Koji Iihara |
| Fukuoka City Hospital | Katsuyuki Hirakawa |
| National Hospital Organization Kyushu Medical Center | Shinji Nagata |
| Saiseikai Fukuoka General Hospital | Akio Ookura |
| Hamanomachi Hospital | Koichirou Matsukado |
| Fukuoka Tokushukai Medical Center | Hidenori Yoshida, Yoshiro Kaneko |
| NHO Fukuoka Higashi Medical Center | Hiroshi Nakane |
| Fukuokaseisyukai Hospital | Isao Inoue |
| Hachisuga Hospital | Yoshihisa Maeda, Kei Hisada |
| Shin Koga Hospital | Tsutomu Hitotsumatsu |
| Omuta City Hospital | Terukazu Kuramoto, Kouichi Kuramoto |
| Kawasaki Hspital | Junya Hayashi |
| Seihokai Tanushimaru Chuo Byoin | Yoshihisa Matumoto, Hiromichi Ooishi |
| Fukuoka University Hospital | Toru Inoue, Masani Nonaka |
| Kurume University Hospital | Motohiro Morioka |
| Kitakyushu Municipal Medical Center | Masahiro Mizoguchi, Haruhisa Tsukamoto |
| Saga-Ken Medical Centre Koseikan | Hiroshi Sugimori, Shuji Sakata, Hiroshi Takashima |
| Yayoigaoka Kage Hospital | Shin-Ichiro Ishihara |
| Karatsu Red Cross Hospital | Kenji Suzuyama |
| Imari Arita Kyoritsu Hospital | Nobuaki Momozaki |
| NHO Ureshino Medical Center | Masayuki Miyazono |
| Yokohama Shintoshi Neurosurgical Hospital | Masafumi Morimoto, Itaro Hattori, Satoshi Ozaki |
| Yokosuka General Hospital Uwamachi | Nobuo Hirota |
| Yokohama City Minato Red Cross Hospital | Yasunori Takemoto, Yasuhiko Mochimatsu, Makoto Takagi |
| Yokohama stroke and Brain Center | Isao Yamamoto, Kenji Nakayama |
| Yokohama Rosai Hospital |  |
| Chigasaki Municipal Hospital | Yoshinori Uchida, Hiroshi Tanaka |
| Yokohama City University Medical Center | Katsumi Sakata |
| Yokohama City University Hospital | Kawahara Nobutaka |
| Yokohama Sakae Kyosai Hospital | Motohiro Nomura |
| Ushioda General Hospital | Hitoshi Ozawa |
| Kawasaki Saiwai Hospital | Kotaro Tsumura |
| Saiseikai Yokohamashi Tobu Hospital | Makoto Inaba, Michiyuki Maruyama |
| Social Insurance Yokohama Chuo Hospital | Tatsuro Mori |
| Showa University Fujigaoka Hospital | Tomoaki Terada |
| Shonan Kamakura General Hospital | Takahisa Mori |
| Sagamihara Kyodo Hospital | Masato Sugitani |
| St. Marianna University School Of Medicine | Yuichiro Tanaka |
| Yamato Municipal Hospital | Masaru Yamada |
| Tokai University School of Medicine | Mitsunori Matsumae |
| Tomei Atsugi Hospital | Keiichirou Onitsuka |
| National Hospital Organization Yokohama Medical Center | Kosuke Miyahara, Tatsuya Takahashi |
| Yokohama Shin-midori General Hospital | Sumio Endou |
| Saitama City Hospital | Atsuhiro Kojima |
| Ageo Central General Hospital | Hidekazu Takahashi |
| Musashino General Hospital | Hiroyuki Kaidu |
| Koshigaya Municipal Hospital | Akira Tsunoda, Chikashi Maruki |
| Saitama Medical Center | Kyoichi Nomura, Toru Matsui |
| Saitama Medical University Hospital | Takamitsu Fujimaki |
| Saitama Cardiovascular And Respiratory Center | Hidetoshi Ooigawa |
| Kan-Etsu Hospital | Masahiko Tanaka, Masatsugu Uchida |
| Saiseikai Kurihashi Hospital | Hiroshi Wanihuti, Kouichi Katoh |
| Fukaya Red Cross Hospital | Hirochiyo Wada |
| Dokkyo Medical University Koshigaya Hospital | Akio Hyodo |
| Japanese Red Cross Maebashi Hospital | Ken Asakura |
| Isesaki Municipal Hospital | Shigeyoshi Nakajima |
| Institute Of Brain And Blood Vessels Mihara Memorial Hospital | Takao Kanzawa |
| Takasaki General Medical Center | Hideyuki Kurihara |
| Kurosawa Hospital | Sigehiro Ohmori |
| Nishiagatsuma Welfare Hospital | Yoshinao Mitsugi, Hiroshi Kusunoki |
| Kiryu Kosei General Hospital | Satoshi Magarisawa |
| Fuji Heavy Industries Health Insurance Society Ota Memorial Hospital |  |
| Mito Saiseikai General Hospital |  |
| Seirei Memorial Hospital | Shinichi Okabe |
| Tsuchiura Kyodo General Hospital |  |
| Ibaraki Prefectural Central Hospital | Yuji Kujiraoka |
| JA Toride Medical Center | Shin Tsuruoka |
| Ushiku　Aiwa　General　Hospital | Mikihiko Takeshita |
| University Of Tsukuba | Tetsuya Yamamoto, Akira Matsumura |
| Tsukuba Medical Center Hospital | Kazuya Uemura |
| Namegata District General Hospital | Hitoshi Tabata |
| National Hospital Organization Mito Medical Center | Makoto Sonobe |
| Ibaraki Seinan Medical Center Hospital |  |
| Ashikaga Red Cross Hospital |  |
| Saiseikai Ustunomiya Hospital | Masashi Nakatsukasa, Ryoji Yoshida |
| Sano Kousei General Hospital |  |
| Shimotsuga General Hospital | Norifumi Shimoeda |
| Fujii Neurosurgical Hospital | Hideo Kunimine |
| Tochigi Medical Center | Masayuki Ishihara |
| Kurosu Hospital | Mikio Teduka |
| Kyoto City Hospital | Nozomu Murai |
| Takeda Hospital | Waro Taki |
| Saiseikai Kyoto Hospital | Nobukuni Murakami |
| Second Okamoto General Hospital | Minoru Kidooka |
| Kyoto Yamashiro General Medical Center | Yoshihiro Iwamoto |
| Kyoto Second Red Cross Hospital | Hiroshi Tenjin |
| Ayabe City Hospital | Kouji Shiga, Masahiko Takamasu |
| Kyoto Miniren Chuo Hospital | Nobuhito Mori |
| Kosei Hospital | Shigeru Kose |
| Kobe University Hospital | Eiji Kohmura |
| National Hospital Organization Kobe Medical Center |  |
| Kobe Red Cross Hospital | Haruo Yamashita |
| JCHO Kobe Central Hospital | Keigo Matsumoto |
| Shinko Hospital |  |
| Nishikobe Medical Center | Naoya Takeda |
| Hyogokenritu_Nishinomiya_Hospital | Takayuki Sakaki |
| Nishinomiya Kyoritsu Neurosurgical Hospital | Hiroji Miyake |
| Hyogo Prefectural Amagasaki General Medical Center |  |
| Takarazuka City Hospital | Eiichiro Mabuchi |
| The Veritas Hospital | Masayuki Yokota |
| Ohnishi Neurological Center | Hideyuki Ohnishi, Yosihiro Kuga |
| Kakogawa West City Hospital |  |
| Nishiwaki Municipal Hospital | Mitsuru Kimura |
| Hyogo Brain and Heart Center |  |
| Steel Memorial Hirohata Hospital |  |
| Tsukazaki　Hospital |  |
| National Hospital Organization Himeji Medical Center | Osamu Narumi, Masaaki Saiki, Norio Nakajima |
| Japanese Red Cross Society Himeji Hospital |  |
| Ako City Hospital | Minoru Asahi |
| Koritsu Toyooka Byoinkumiairitsu Toyooka Hospital | Junji Koyama |
| Hyogo Prefectual Awaji Medical Center | Yoshio Sakagami |
| Itami Kousei Neurosurgical Hospital | Shinya Noda |
| Nara Prefectural Nara Hospital | Junichi Iida |
| Saiseikai Chuwa Hospital | Toyohisa Fujita |
| Nara Medical University | Hiroyuki Nakase |
| Saiseikai Gose Byouin |  |
| Nation Hospital Organization Nara Medical Center | Hidehiro Hirabayashi, Toru Hoshida |
| Matumotokaiseikai Nishinara Central Hospital | Takayoshi Fujimoto |
| Wakayama Medical University Hospital | Naoyuki Nakao |
| Wakayama Medical University Kihoku Hospital | Yoshiyuki Tanaka |
| Hidaka General Hospital | Fuminori Ozaki |
| Minami Wakayama Medical Center | Yoshinari Nakamura |
| Shingu Municipal Medical Center | Kazuhito Miki |
| Wakayama Seikyo Hospital | Teruyuki Habu |
| Tottori University | Takashi Watanabe |
| Yonago Medical Center |  |
| Hirosaki University School Of Medicine And Hospital | Hiroki Ohkuma |
| Kuroishi General Hospital | Seiko Hasegawa |
| Japanese Red Cross Society Hachinohe Hospital | Hiromu Konno |
| Aomori City Hospital | Atsuhito Takemura |
| Odate Municipal General Hospital | Atsuya Okubo |
| Akita　City　Hospital | Hitoshi Saito |
| Research Institute For Brain And Blood Vessels - Akita | Tatsuya Ishikawa, Taizen Nakase |
| Akita University Hospital | Hiroaki Shimizu, Toshio Sasajima |
| Kazuno Kosei Hospital | Masayuki Sasou |
| Fukushima Red Cross Hospital | Yoichi Watanabe |
| Fukushima Medical University Hospital | Taku Sato, Kiyoshi Saito |
| Fujita General Hospital | Satoshi Taira, Masahiro Satoh |
| Southern Touhoku General Hospital | Zenichiro　Watanabe |
| Takeda General Hospital | Takayuki　Koizumi |
| Iwaki Kyoritsu General Hospital | Yasuhiro Suzuki, Shoji Mashiyama |
| Minamisouma City General Hospital | Tomoyoshi Oikawa |
| Yamagata University Hospital | Yukihiko Sonoda |
| Yamagata City Hospital Saiseikan | Rei Kondo, Shinjiro Saito |
| Shinoda General Hospital | Atsuo Shinoda |
| Kitamurayama Hospital | Eiichiro Kamatsuka |
| Yamagata Prefectural Shinjo Hospital | Keiten So |
| Okitama Public General Hospital | Toshihiko Kinjo |
| Yonezawa City Hospital | Toru Sasaki, Kenji Ito |
| Sanyudo Hospital | Yohei Kudoh |
| Tsuruoka Municipal Shonai Hospital | Kazuhiko Sato |
| Kohnan Hospital | Hidenori Endo, Hiroaki Shimizu |
| Sendai City Hospital | Hiroshi Karibe |
| Furukawaseiryou Byouin | Kou Takahashi |
| Omihachiman Community Medical Center | Masayuki Nakajima |
| Kohka Public Hospital | Kazuyoshi Watanabe |
| Shiga University Of Medical Science | Kazuhiko Nozaki |
| Otsu Municipal Hospital | Motohiro Takayama |
| Nagahama City Hospital | Taro Komuro |
| Koto Memorial Hospital | Hisao Hirai, Fumio Suzuki |
| Yokkaichi Municipal Hospital |  |
| Mie University Hospital | Hidenori Suzuki |
| Saiseikai Matsusaka General Hospital | Hiroto Murata |
| Ise Red Cross Hospital | Fumitaka Miya |
| Kuwana West Medical Center |  |
| Suzuka Kaisei Hospital | Kenji Kanamaru |
| Fuji Brain Institute Hospital | Akira Tamura |
| Shizuoka General Hospital | Kiyoshi Harada |
| Shizuoka Municipal Hospital | Seiji Fukazawa |
| Yaizu City Hospital | Seiya Takehara |
| Hamamatsu Rosai Hospital Japan Labour Health and Welfare Organization | Yoshihiko Watanabe |
| Hamamatsu Medical Center | Teiji Nakayama |
| Seirei Mikatahara General Hospital | Haruhiko Sato, Hiroshi Nagura |
| Fukuroi Municipal Hospital |  |
| Iwata Municipal General Hospital | Shinji Amano, Chiharu Tanoi |
| National Hospital Organization Shizuoka Medical Center | Katsuhiro Kuroda |
| Fuji City General Hospital | Satoru Morooka |
| Shizuoka Children's Hospital | Takafumi Wataya, Masashi Kitagawa |
| Kakegawa Municipal General Hospital | Kazuo Koide |
| Gifu Municipal Hospital | Tetsuya Tanigawara |
| Gifu University Hospital | Toru Iwama |
| Gifu Prefectural Tajimi Hospital | Junki Ito |
| Toki General Hospital | Shinji Noda |
| Saku Central Hospital | Kazuyuki Kohno |
| Aizawa Hospital | Kazuo Kitazawa |
| Nagano Municipal Hospital | Yoshikazu Kusano, Toshiki Takemae |
| Nagano Prefectural Suzaka Hospital |  |
| Shinonoi General Hospital | Masanobu Hokama |
| Suwa Central Hospital | Hiroki Sato, Yoshihisa Nishiyama |
| Seguchi Neuro Surgery Hospital | Tatsuya Seguchi |
| Iida Municipal Hospital | Sumio Kobayashi, Yoshihiko Inui, Yoji Ohigashi |
| Showa Inan General Hospital | Shinsuke Muraoka |
| Japanese Red Cross Society Azumino Hospital | Masaki Miyatake |
| Azumi General Hospital | Kensuke Hayashida, Shinichi Nakagawa |
| Nagano Prefectural Kiso Hospital | Atsushi Inoue |
| Nho Shinshu Ueda Medical Center | Keiichi Sakai |
| Shimane University Hospital | Shuhei Yamaguchi |
| Shimane Prefectural Central Hospital | Tatsuya Mizoue, Fusao Ikawa |
| Yasugi Municipal Hospital | Gen Ishida, Hideki Irie |
| National Hospital Organization Hamada Medical Center | Takato Kagawa |
| National Hospital Organization Okayama Medical Center | Yoichiro Namba |
| Okayama Kyokuto Hospital | Hiroyuki Nakashima |
| Okayama City Hospital | Koji Tokunaga |
| Okayama University Hospital | Isao Date, Koji Abe |
| Kawasaki Medical School Hospital | Masaaki Uno |
| Kurashiki Central Hospital | Masaki Chin, Sen Yamagata |
| Kurashiki-Heisei Hospital | Hidemichi Sasayama, Soichiro Takao |
| Tsuyama Chuo Hospital | Hideyuki Yoshida, Koji Muneda |
| Okayama Kyoritsu General Hospital |  |
| Mizushima Central Hospital |  |
| Kasaoka Daiichi Hospital | Akira Watanebe |
| Tokuyama Central Hospital | Kunihiko Harada |
| Saiseikai Yamaguchi General Hospital | Syouichi Kato |
| Yamaguchi Prefectural Grand Medical Center |  |
| Japanese Red Cross Yamaguchi Hospital | Yasuhiro Hamada |
| Department Of Neurosurgery, Yamaguchi University School Of Medicine | Michiyasu Suzuki |
| Ube Industries Central Hospital | Takafumi Nishizaki |
| Kanmon Medical Center | Katsuhiro Yamashita |
| Shimonoseki City Hospital | Takaharu Nakamura |
| Shimonoseki Medical Center |  |
| Hiroshima Red Cross Hospital & Atomic-Bomb Survivors Hospital |  |
| Suiseikai Kajikawa Hospital | Shinichi Wakabayashi |
| Hiroshima University Hospital | Takahito Okazaki, Kaoru Kurisu, Masayasu Matsumoto, Naohisa Hosomi |
| Hiroshima Prefectural Hospital | Atsushi Tominaga, Katsuzo Kiya |
| Araki Neurosurgical Hospital | Masaaki Shibukawa, Syuichi Oki |
| Hiroshima City Asa Hospital |  |
| Itsukaichi Memorial Hospital |  |
| Mazda Hospital | Toshinori Nakahara |
| Chugoku Rosai Hospital | Shinji Okita |
| National Hospital Organization Kure Medical Center | Tsuyosi Torii |
| Kure Kyosai Hospital |  |
| Rijinkai Medical Foundation Socio-Medical Corporation Kohsei General Hospital | Minoru Nakagawa, Kenjirou Fujiwara |
| Mitsugi General Hospital | Takashi Matsuoka, Syuhei Nishimura |
| Brainattack Center Oota Memorial Hospital |  |
| Miyoshi Central Hospital | Osamu Hamasaki, Naoyuki Isobe |
| Tokushima University | Junichiro Satomi, Shinji Nagahiro |
| Tokushima Prefecture Naruto Hospital | Masahito Agawa |
| Tokushima Prefectural Kaifu Hospital | Hirofumi Oka |
| Houetsu Hospital |  |
| Kansai Medical University Hirakata Hospital | Kunikazu　 Yoshimura |
| Japan Labor Health Welfare Organization Kyushu Rosai Hospital | Sei Haga |
| Teinekeijinkai Hospital | Katsuyuki Asaoka |
| Sapporo Azabu Neurosurgical Hospital | Toshitaka Nakamura |
| Hakodate Central General Hospital | Tsutomu Kato |
| Hokusyoukai Otaru Chuo Hospital | Nobuaki Kobayasi, Satoshi Minoshima |
| Sapporo Medical University Hospital | Nobuhiro Mikuni |
| Hakodate Municipal Hospital | Jun Niwa |
| Sapporo Teishinkai Hospital | Rokuya Tanikawa |
| Shinsapporo Neurosurgical Hospital | Akinori Yamamura |
| Sendai East Neurosurgical Hospital | Noriaki Watabe |
| JA Akita Kouseiren Oomagari Kousei Medical Center | Jyunkou Sasaki |
| Noshiro Kosei Medical Center | Yasunari Otawara |
| Iwate Prefectural Kuji Hospital | Kazuyuki Miura |
| Tohoku University Hospital | Teiji Tominaga |
| Aomori Prefectural Central Hospital | Tatsuya Sasaki |
| Iwate Prefectural Central Hospital | Takayuki Sugawara |
| NHO Sendai Medical Center | Masayuki Ezura |
| Ishinomaki Red Cross Hospital | Syuichi Ishikawa |
| Social Welfare Organization Saiseikai Imperial Gift Foundation Inc.Yamagata Saisei Hospital | Sunao Takemura |
| Ohta Nishinouchi Hospital | Masahisa Kawakami |
| Tokyo Metropolitan Childrens Medical Center | Satoshi Ihara |
| Mito Kyodo General Hospital | Yasushi Shibata |
| Chiba Rosai Hospital | Takashi Saegusa |
| Chiba Cancer Center | Toshihiko Iuchi |
| Chiba Children's Hospital | Chiaki Ito |
| Saiseikai Kawaguchi Hospital | Sumio Isimaru |
| Juntendo Tokyo Koto Geriatric Medical Center | Osamu Okuda |
| School Of Medicine Keio University | Kazunari Yoshida |
| Jinmeikai Akiyama Hospital of Neurosurgery and Internal Medicine | Takekazu Akiyama |
| Tokyo Dental College Ichikawa General Hospital | Sadao Suga, Masateru Katayama |
| Mitsuwadai General Hospital | Masahiko Kasai |
| Ebina General Hospital |  |
| Tokyo Metropolitan Health and Medical Treatment Corporation Ohkubo Hospital | Akihiro Oikawa |
| Itabashi Chuo Medical Center | Naohisa Miura |
| Tokyo Metropolitan Tama Medical Center | Takahiro Ota |
| Teraoka Memorial Hospital | Atsumi Takenobu |
| Kitasato University School Of Medicine | Toshihiro Kumabe |
| Yokohama Asahi Chuo General Hospital | Sachio Suzuki |
| Yamagata Prefectural Central Hospital | Takashi Kumagai |
| Japanese Red Cross Akita Hospital | Keiichi Nishimaki |
| Shinshu University Hospital | Kazuhiro Hongo |
| Nagano Children's Hospital | Hiroaki Shigeta |
| Ina Central Hospital | Atsushi Sato |
| Toyama University Hospital | Satoshi Kuroda |
| Keiju Medical Center | Sotaro Higashi |
| Toyohashi Municipal Hospital | Hirofumi Oyama |
| Chubu Rosai Hospital | Kazuyoshi Hattori |
| Kamiiida Daiichi General Hospital | Yoichi Uozumi |
| Nagoya Central Hospital | Norimoto Nakahara |
| Nagoya City East Medical Center | Nobukazu Hashimoto |
| Chutoen General Medical Center | Toshikazu Ichihashi |
| Japanese Red Cross Takayama Hospital | Katsunobu Takenaka, Yuko Nonaka |
| Daiyukai General Hospital | Shinichi Shirakami, Shu Imai |
| Seikeikai Hospital | Yoshinari Okumura |
| Higashiosaka City General Hospital | Ryo Tamaki, Kazuhiro Yokoyama |
| Kyoto University Graduate School Of Medicine | Susumu Miyamoto |
| Tenri Hospital | Yoshinori Akiyama |
| Kishiwada City Hospital | Kenji Hashimoto |
| Rakuwakai Otowa Hospital | Kazuo Yamamoto |
| First Towakai Hospital | Tsugumichi Ichioka |
| Kano Hospital | Kazutomo Nakazawa |
| Moriguchi-Ikuno Memorial Hospital | Misao Nishikawa |
| Ishikiriseiki Hospital | Tsuyoshi Inoue |
| Osaka Medical Center for Cancer and Cardiovascular Diseases | Manabu Kinoshita |
| Rinku General Medical Center |  |
| Hyogo College Of Medicine | Shinichi Yoshimura |
| Akashi City Hospital | Minoru Saitoh |
| Hyogo Prefectural Kakogawa Medical Center | Hideo Aihara |
| Tokushima Red Cross Hospital | Hajimu Miyake |
| National Hospital Organization, Iwakuni Clinical Center | Kotaro Ogihara, Tsukasa Nishiura |
| Hiroshima City Hiroshima Citizens Hospital | Shigeki Nishino |
| Okayama Saiseikai General Hospital | Yasuyuki Miyoshi |
| Fukuyama City Hospital | Tadashi Arisawa |
| Onomichi Municipal Hospital | Shigeru Daido, Shoji Tsuchimoto |
| Kaneda Hospital | Kimihisa Kinoshita |
| Higashihiroshima Medical Center | Kiyoshi Yuki, Keisuke Migita |
| Tottori Municipal Hospital | Keiichi Akatsuka |
| Shuto General Hospital | Hirosuke Fujisawa |
| University Of Occupational And Environmental Health | Junkoh Yamamoto |
| Japan Community Health Care Organization Kyushu Hospital | Satoshi Inoha |
| Kyushu Central Hospital Of The Mutual Aid Association Of Public School Teachers | Hitonori Takaba |
| Harasanshin Hospital | Tadahisa Shono |
| Japanese Red Cross Fukuoka Hospital | Hitoshi Tsugu |
| Hakujyuji Hospital | Shuji Hayashi |
| School Of Medicine, Saga University | Tatsuya Abe, Toshio Matsushima |
| St. Mary's Hospital | Susumu Nakashima |
| Tobata Kyoritsu Hospital | Takehisa Tuji |
| JCHO Hitoyoshi Medical Center | Keizo Yamamoto |
| Koseiren Tsurumi Hospital | Akihiko Kaga |
| Sanseikai Kanemaru Neurosurgery Hospitaｌ | Reizou Kanemaru |
| Atsuchi Neurosurgical Hospital | Koji Takasaki |
| Kagoshima Medical Center | Junichi Imamura |
| Okinawa Red Cross Hospital | Masahiro Noha |
| Asahi General Hospital | Saburo Watanabe |
| Kobe City Medical Center General Hospital | Nobuyuki Sakai |
| Yoshida Hospital.Cerebrovascular Research Institute | Yasuhisa Yoshida, Hiroaki Minami |
| Teishinkai Hospital | Tomoyoshi Okumura |
| Southern Tohoku General Hospital | Shinjitsu Nishimura |
| Tokyo General Hospital | Shinichi Numazawa |
| St. Luke's International Hospital | Yasunari Niimi |
| Fukuoka University Chikushi Hospital | Kiyoshi Kazekawa, Masanori Tsutsumi |
| Fukuoka Wajiro Hospital | Kouzou Fukuyama |
| Shintakeo Hospital | Makoto Ichinose |
| Kansai Electric Power Hospital | Yasuhiro Fujimoto |
| Tomakomai Higashi Hospital | Youichi Hashimoto |
| Date Red Cross Hospital | Takeshi Matsuoka |
| Hirosaki Stroke And Rehabilitation Center | Takamitsu Uchizawa |
| Kitakami Saiseikai Hospital | Tomohiko Sato |
| Kamagaya General Hospital | Hiroaki Sawaura |
| Higashitotsuka Memorial Hospital | Satoshi Utsuki |
| National Hospital Organization Niigata National Hospital | Chiaki Takahashi |
| Toyama Red Cross Hospital | Kazumasa Yamatani |
| Maruko Central Hospital | Toshiyuki Tsukada |
| Okaya City Hospital | Ryoichi Hayashi |
| Kenwakai Hospital | Masakazu Kitahara |
| Suwa Red Cross Hospital | Yukinari Kakizawa |
| Kyoto-Katsura Hospital | Yasumasa Yamamoto |
| Shimizu Hospital | Takashi Yoshida |
| Social Welfare Organization Saiseikai Imperial Gift Foundation Inc.Osaka Saiseikai Ibaraki Hospital | Yasunobu Goto |
| Kobe Ekisaikai Hospital | Takashi Tominaga |
| Kitaharima Medical Center | Shigeru Miyake |
| Fujii Masao Memorial Hospital | Nozomi Mori |
| HITO Medical Center | Naoki Shinohara |
| Chidoribashi Hospital | Yasushi Ejima |
| Souseikai Shin Yoshizuka Hospital | Mayumi Mori |
| Miyake Neurosurgical Hospital | Hitoshi Miyake |
| Nakatsu Municipal Hospital | Hiromichi Koga, Kenichi Matsumoto |
| Nakatsu Neurosurgical Hospital | Kazuya Morimoto |
| Otaru General Hospital | Yoshimasa Niiya |
| Shuuwa General Hospital | Tsuneo Shishido |
| Fukuchiyama City Hospital | Mamoru Murakami |
| Saku Central Hospital Advanced Care Center | Takaaki Yoshida |
| Inazawa Municipal Hospital | Masahito Hara |
| Nishitokyo Central General Hospital | Tatsuya Nakamura |
| Koyama Memorial Hospital | Takuya Kawai |
| The Foundation Shinwakai Yachiyo Hospital | Takashi Inoue |
| Ainomiyako Neurosurgery Hospital | Isao Sasaki |
| Fukuokaken Saiseikai Futsukaichi Hospital | Naoko Fujimura |
